# Nursing Progress and Its Developments: IX. The Local Centres of the College of Nursing

**Published:** 1918-03-09

**Authors:** 


### THE MATRONS' AND SISTERS' DEPARTMENT.

### NURSING PROGRESS AND ITS DEVELOPMENTS.

### IX.—The Local Centres of the College of Nursing.

IT is characteristic of British methods to decentralise. British people are wont to rebel against the mere suspicion of being governed by a bureaucracy, which is literally management under officials appointed from a central office or bureau. They prefer to appoint their own officials and manage their own affairs. Hence the College of Nursing has done wisely in encouraging from the outset the principle of local centres administered in the interests of members by members themselves chosen in each town or centre by the nurses residing within the area. The formation of centres in Liverpool and Manchester marks a very important stage in the evolution of a representative College of Nursing.

Before considering in what manner nurses may benefit themselves by forming autonomous groups in various parts of the country, it may be well to inquire what benefits may be expected to accrue to the central organisation of the College of Nursing through the medium of its local centres. Some anxious souls may be ready to imagine a danger in loss of unity, or division of control, as a result of local self-government among the members. The main principle, however, for which the College is contending, State Registration of Nurses, with a one-portal examination, is accepted by all members when they join. The College is at present a militant body which has embarked on a campaign to redress certain abuses in the nursing profession, only to be satisfactorily swept away by an Act for the State Registration of Nurses. Whatever may ultimately prove the scope of the College, its immediate policy is to press forward with all sails set towards the goal of State Registration. It has always been abundantly clear that Parliament will lend a willing ear to the united voice of any profession which desires to regulate itself in accordance with the interests of the public. How, then, can the united voice of the nursing profession best make itself heard? Solely through the agency of nurses grouped together in centres under the ægis of the College in every part of the country.

The College of Nursing as a central body in London can command large and representative meetings whenever it desires. But at these meetings possibly but four or five nurses respectively may be able to attend from Liverpool, Manchester, Birmingham, and the other big towns. A resolution in favour of registration may be passed at such a meeting and may record the unanimous desire of three or four hundred nurses to organise their profession on sound lines. But provincial members could only read about it in the papers; they would feel outsiders. Now suppose there were a hundred meetings in different parts of the country recording the same resolution, who does not see that the voice of the nurses would unmistakably have made itself heard in each locality? The voices in favour of State Registration number not merely a few hundreds, but many thousands. The first great duty of each local centre, therefore, towards the College is so to organise itself that every member may be enabled to record her individual vote in favour of State Registration as and when she may be asked to do so. The College ought to be able to count, when the moment comes for impressing the House of Commons, on an enthusiastic outburst of opinion, spontaneously proffered by nurses, linked together in their own centres in every part of the kingdom under officers chosen by themselves.

We have laid stress on this aspect of the local centre because the very existence of the College is an earnest of the great impending struggle to obtain State recognition for nurses.

But the establishment of strong centres is necessary to the life of the College because they are the natural sources through which the College can be fed and strengthened by a mighty accession of numbers and influence. There is a continuous drain on every body of nurses from other causes besides death. Members marry and move away, and their identity is lost. They go forth to every part of the Empire, on every kind of business, and respond no longer, it may be for years, nor furnish any address. The nursing profession is like a mighty column of troops slowly defiling across a mountain pass into a land of promise. From the local centre, stretching out, as it were, in a boundless plain below, must come the needful and ceaseless accession of recruits to bring this great army to secure victory. Not for their own interests alone is the battle to be waged. Sickness, suffering, poverty, and vice are waiting at every turn of the road, and must be met and conquered before the goal is reached. The aims towards which the nursing profession, once it has been well organised under the recognition of the State, shall press forward lie in very truth beyond the clouds.

